# Pt/WO_3_ Nanoparticle-Dispersed Polydimethylsiloxane Membranes for Transparent and Flexible Hydrogen Gas Leakage Sensors

**DOI:** 10.3390/membranes12030291

**Published:** 2022-03-02

**Authors:** Ryo Ishihara, Yoshihiro Makino, Yuki Yamaguchi, Kenjiro Fujimoto, Keishi Nishio

**Affiliations:** 1 Department of Materials Science and Technology, Tokyo University of Science, 6-3-1 Niijuku, Katsushika, Tokyo 125-8585, Japan; r-ishihara@juntendo.ac.jp (R.I.); 8216656@alumni.tus.ac.jp (Y.M.); 2 Department of Pure and Applied Chemistry, Tokyo University of Science, Noda 278-8510, Japan; y-yamaguchi@aist.go.jp (Y.Y.); fujimoto_kenjiro@rs.tus.ac.jp (K.F.)

**Keywords:** hydrogen gas sensor, polydimethylsiloxane, transparent, flexible, platinum-catalyst-loaded tungsten trioxide

## Abstract

Hydrogen gas is a promising, clean, and highly efficient energy source. However, to use combustible H_2_ gas safety, high-performance and safe gas leakage sensors are required. In this study, transparent and flexible platinum-catalyst-loaded tungsten trioxide (Pt/WO_3_) nanoparticle-dispersed membranes were prepared as H_2_ gas leakage sensors. The nanoparticle-dispersed membrane with a Pt:W compositional ratio of 1:13 was transparent and exhibited a sufficient color change in response to H_2_ gas. The membrane containing 0.75 wt.% of Pt/WO_3_ nanoparticles exhibited high transparency over a wide wavelength range and the largest transmittance change in response to H_2_ gas among the others. The heat treatment of the particles at 573 K provided sufficient crystallinity and an accessible area for a gasochromic reaction, resulting in a rapid and sensitive response to the presence of H_2_ gas. The lower limit of detection of the optimized Pt/WO_3_ nanoparticle-dispersed membrane by naked eye was 0.4%, which was one-tenth of the minimum explosive concentration. This novel membrane was transparent as well as flexible and exhibited a clear and rapid color response to H_2_. Therefore, it is an ideal candidate sensor for the safe and easy detection of H_2_ gas leakage.

## 1. Introduction

Hydrogen gas is expected to emerge as a next-generation energy source because of its high energy density and low environmental load compared with fossil fuels [1]. However, it is a transparent and odorless gas that poses a risk of explosion when its concentration in the atmosphere is in the approximate range of 4–74 vol%. In addition, hydrogen has a relatively high diffusion coefficient compared with other combustible gases. Therefore, gas leakage detection sensors are essential for its safe utilization. Current semiconductor-type and contact combustion-type hydrogen sensors have a disadvantage of requiring high operating temperatures and heating processes. They cannot be used when power failures occur because a power supply would be required to detect the changes in the electrical resistance of the elements. Moreover, it is desirable that the sensors can be used for leakage detection in transportation pipes and storage tanks with complicated shapes.

Platinum-loaded tungsten trioxide (Pt/WO_3_) exhibits a rapid and reversible color change (known as gasochromism) through redox reactions with hydrogen and oxygen, even at room temperature [2,3,4]. The gasochromism of Pt/WO_3_ consists of four steps: (i) decomposition of hydrogen molecules to hydrogen atoms on the Pt catalyst, (ii) ionization of the produced hydrogen via diffusion toward the WO_3_ particles, (iii) diffusion of protons and electrons into the WO_3_ structure, which is driven by the concentration gradient, and (iv) excitation of electrons trapped in the d-orbital of W and WO_3_ by visible light, leading to a blue color. Consequently, Pt/WO_3_ allows a rapid and inexpensive detection of the leakage of the colorless and odorless hydrogen gas by the naked eye. Moreover, as no electricity is used, we can determine the presence of hydrogen using the color change even after a power failure. Therefore, Pt/WO_3_ is a promising material for fabricating hydrogen sensors.

However, Pt/WO_3_ nanoparticles cannot be handled easily when they are used by themselves. Consequently, they have been utilized in the form of metal oxide thin films on the surfaces of heat-resistant substrates, such as glass [5,6] and alumina [7]. This is because the conventional preparation of the Pt/WO_3_ nanoparticles involves a heating process at 700–800 K. However, as these substrates are not flexible, they cannot be easily attached to transportation pipes and storage tanks with complicated shapes. Recently, flexible polymers have been used as substrate materials for the fabrication of Pt/WO_3_ particle thin films under mild conditions using a sol-gel method [8,9,10,11,12]. Although methods for synthesizing WO_3_ particles under mild conditions have already been reported (e.g., a hydrothermal method at 350 K [13]), the sol-gel process can synthesize WO_3_ nanoparticles and simultaneously load the Pt catalyst, producing a large number of nanoparticles compared with hydrothermal methods. Furthermore, the Pt/WO_3_ particles prepared using the sol-gel method possess a mixture of crystalline and amorphous phases, which has the advantage of the easy penetration of the particles by hydrogen, thereby shortening the time required for color change. Additionally, crystallinity can be conveniently controlled using the heat treatment conditions of the particles. For both previous and sol-gel methods, Pt/WO_3_ layers were formed on the substrate materials after the substrates had already been produced. In addition, there is a lack of studies on the aging degradation of Pt/WO_3_ layers on the substrate surfaces.

In this study, a novel fabrication process of hydrogen sensor based on Pt/WO_3_ nanoparticles and a polymer is proposed. In this process, instead of forming Pt/WO_3_ layers on the surface of existing substrates, Pt/WO_3_ particles are prepared in advance and are then incorporated into a polymer membrane synthesis process. Accordingly, we can use flexible polymer materials without concerns about temperatures when fabricating Pt/WO_3_. The Pt/WO_3_ nanoparticles, prepared in advance, were dispersed in a precursor solution to synthesize a polymer substrate and obtain a Pt/WO_3_ nanoparticle-dispersed membrane. The Pt/WO_3_ nanoparticles exist not only on the surface but are also distributed and exhibit a color change throughout the material. They are expected to be resistant to aging degradations, such as abrasion. Polydimethylsiloxane (PDMS) membrane, which is transparent, flexible, and gas-permeable, was selected as the polymer. Because of its characteristics, PDMS membranes have been applied in many research fields [14,15,16,17,18,19]. Here, the optimum heat treatment temperature, content, and crystal structure of Pt/WO_3_ nanoparticles have been investigated. Finally, a novel flexible, transparent, resistant, and cost-efficient hydrogen gas leakage membrane sensor was developed to benefit from the rapid and reversible gasochromism of Pt/WO_3_ nanoparticles to H_2_ gas.

## 2. Materials and Methods

### 2.1. Materials

Tungsten hexachloride (WCl_6_) and hydrogen hexachloroplatinate hexahydrate (H_2_PtCl_6_·6H_2_O) for the PtWO_3_ nanoparticles were purchased from Kanto Chemical Co., Inc. (Tokyo, Japan) and Kishida Chemical Co., Ltd. (Osaka, Japan), respectively. Ethanol was purchased from Kanto Chemical Co., Inc. (Tokyo, Japan). Tertiary butyl alcohol was purchased from Wako Pure Chemical Industries, Ltd. (Osaka, Japan). Sylgard^®^ 184 silicone elastomer kit for the membrane base was purchased from Dow Corning Co. (Midland, MI, USA).

### 2.2. Synthesis of Pt-Particle-Dispersed Tungsten Trioxide (Pt/WO_3_) Nanoparticles

Tungsten hexachloride and hydrogen hexachloroplatinate hexahydrate were first dissolved in ethanol at predetermined atomic ratios (Pt:W = 1:13, 1:100, 1:1000) to form Pt/WO_3_ precursor solutions in a dry nitrogen atmosphere. These solutions were then dried in the air at 393 K for 12 h. Next, the dried residues were heated at 473–773 K for 1 h to obtain Pt/WO_3_ powders. The crystal structures and crystallinities of the obtained powders were characterized using X-ray diffraction (XRD, Ultima IV, Rigaku Corporation, Tokyo, Japan).

### 2.3. Preparation of Pt/WO_3_ Nanoparticle-Dispersed Polydimethylsiloxane Membranes

The prepared Pt/WO_3_ powders were first bead-milled with *t*-butyl alcohol and zirconia beads at 1500 rpm for 1 h. The obtained suspensions were then centrifuged twice at 4800 rpm for 30 min to prepare Pt/WO_3_ nanoparticle dispersions. These dispersions were added to Sylgard^®^ 184 base at concentrations ranging from 0.50 to 3.00 wt.% and stirred at 393 K and 200 rpm to prepare Pt/WO_3_ nanoparticle-dispersed bases. Finally, a curing agent (Sylgard^®^ 184 Silicone Elastomer Curing Agent, Dow Corning, Midland, MI, USA) was added to the bases at 10 wt.%. The mixtures were subsequently degassed for 30 min and heated at 353 K for 2 h to obtain Pt/WO_3_ nanoparticle-dispersed PDMS membranes.

### 2.4. Evaluation of Hydrogen Gas Response of Pt/WO_3_ Composite Membranes

**Figure 1:**
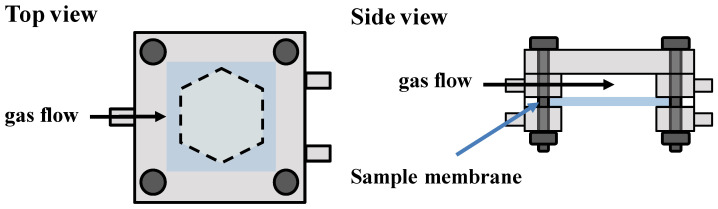
Experimental apparatus for evaluating H_2_ gas response of membranes.

The optical response of the Pt/WO_3_ nanoparticle-dispersed PDMS membranes to hydrogen gas was evaluated using ultraviolet-visible spectroscopy (UV-630, Jasco, Tokyo, Japan). The prepared membranes were placed in a transparent acrylic cell (Figure 1). Baseline correction was performed using an empty cell. The transmission spectrum of the membranes was measured from 400 to 1100 nm by exposing one side of the membrane to synthetic air or 100% H_2_ gas. The absorbance at 800 nm was measured to evaluate their hydrogen gas-sensing performance. A PDMS membrane containing Pt/WO_3_ nanoparticles (0.75 wt.% of Pt:W = 1:13) heat treated at 573 K was used for low-concentration H_2_ gas detection (H_2_ was diluted with synthetic air). The absorbance was normalized as follows:

ΔAbs = Abs − Abs_0_ (1)

where Abs and Abs_0_ are the absorbances at time *t* and 0 s, respectively. The value of *t* ranged from 0 to 600 s. The color difference, transmittance change (ΔT), was calculated as follows:

ΔT = transmittance in air at 800 nm − transmittance in H_2_ at 800 nm (2)

## 3. Results and Discussion

### 3.1. Effect of Compositional Ratio of Platinum and Tungsten Trioxide

**Figure 2:**
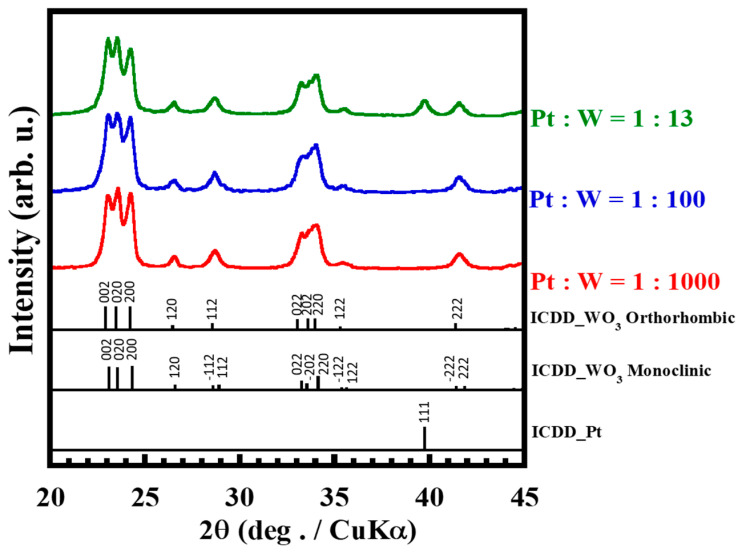
XRD spectra of Pt/WO_3_ nanoparticles with different amounts of the Pt catalyst.

The XRD analysis results for the powders with different amounts of Pt are shown in Figure 2. All powders were dried at 393 K and calcined at 673 K for 1 h. The diffraction patterns of the powders correspond to the peaks of monoclinic WO_3_ (ICDD 01-072-0677) and orthorhombic WO_3_ (ICDD 01-071-0131). Focusing on the diffraction peak around 29°, the shoulder was observed at higher angle. This was attributed to monoclinic phase as shown in the reference. In contrast, the broad peaks were observed around 44°. These peaks were attributed to orthorhombic phase. No difference in crystallinity was observed between the samples. The diffraction pattern of Pt (ICDD 00-004-0802) is present only in the diffractogram of the Pt:W = 1:13 powder sample. Therefore, the prepared Pt/WO_3_ powders were a mixed phase of monoclinic and orthorhombic crystals. The crystallinity did not depend on the amount of loaded Pt.

**Figure 3:**
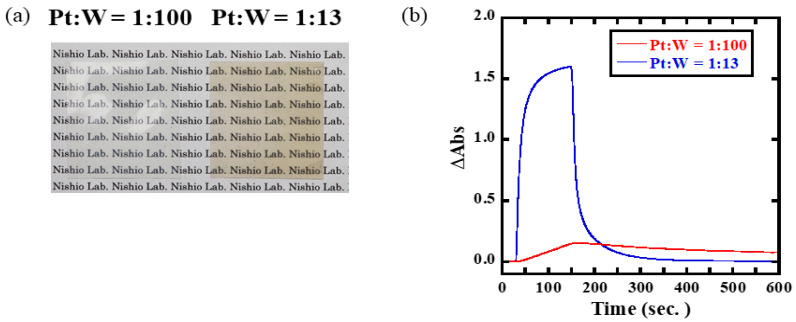
Dependence of H_2_ response on the amount of the Pt catalyst in Pt/WO_3_ nanoparticles. (**a**) Typical image of Pt/WO_3_ nanoparticle-dispersed membranes. (**b**) H_2_ response measured at 800 nm.

A photograph of the membranes prepared using the powders under atmospheric conditions is shown in Figure 3a. The Pt/WO_3_ particle content of the membranes was 0.75 wt.%. Both sample membranes were transparent. A slight coloration was observed in the sample with higher Pt loading (Pt:W = 1:13). This originates from the presence of Pt black. The sizes of Pt particles impregnated on Pt/WO_3_ prepared by the sol-gel method have been reported to be approximately 5–20 nm, which correspond to those of Pt black [5]. The particle sizes of WO_3_ in the Pt:W = 1:13 and Pt:W = 1:100 powder samples were 41.0 ± 7.2 nm and 46.2 ± 6.3 nm, respectively (Figure S1 in Supplementary Materials). These results agree well with our previous report [20].

The transmission spectra of the sample membranes and time variation of the absorbance after exposure to 100% H_2_ gas are shown in Figure S2 and Figure 3b, respectively. The membrane with the higher Pt loading (Pt:W = 1:13) exhibited a faster response to H_2_ gas than the one with a lower Pt loading (Pt:W = 1:100). This is because the higher loading in the former provides a higher probability of the Pt contact with hydrogen molecules, which results in the faster atomization of hydrogen molecules. Considering these results, the Pt/WO_3_ membrane with the higher loading of Pt nanoparticles (Pt:W = 1:13) was selected for subsequent experiments.

### 3.2. Optimization of Pt/WO_3_ Nanoparticle Content

**Figure 4:**
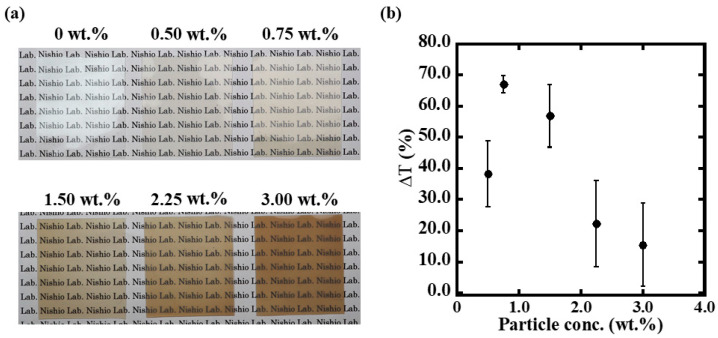
Dependence of transparency and H_2_ response on the percentage content of Pt/WO_3_ nanoparticles. (**a**) Typical images of sample membranes. (**b**) Transmittance change when the films were exposed to 100% H_2_ gas measured at 800 nm.

Photographs of the membranes containing 0.00–3.00 wt.% of Pt/WO_3_ under ambient conditions are shown in Figure 4a. Pt/WO_3_ particles were prepared by calcining the Pt:W = 1:13 powder at 673 K. The transparency decreased, and the color changed to dark brown as the number of particles dispersed in the membranes increased. Transmittance at 800 nm is shown in Figure S3a (Supplementary Materials).

To investigate the effect of hydrogen gas coloration on the number of dispersed particles, the transmission spectra of membranes exposed to synthetic air and 100% H_2_ gas are shown in Figure S3b (Supplementary Materials). The color difference response to H_2_ gas reached the local maximum value of 67.0 ± 2.7% in the 0.75 wt.% Pt/WO_3_ membrane and decreased at higher particle contents. The 0.75 wt.% Pt/WO_3_ membrane showed high transparency over a wide wavelength range. The color difference at 800 nm is plotted in Figure 4b. The 0.75 wt.% Pt/WO_3_ membrane showed the largest transmittance change at 800 nm while there was a sufficient amount of Pt particles for coloring. Considering its applicability for naked-eye hydrogen gas leakage detection, 0.75 wt.% Pt/WO_3_ was selected as the particle content for the subsequent experiments.

### 3.3. Optimization of Heat Treatment Temperature on Hydrogen Gas Response Time

**Figure 5:**
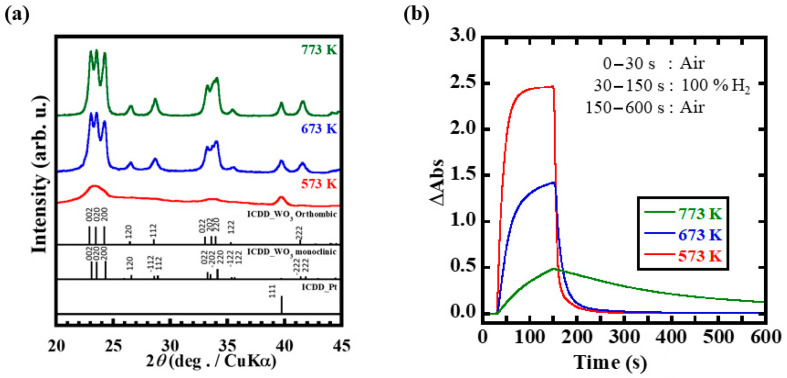
Dependence of H_2_ response on the Pt/WO_3_ nanoparticle heat treatment temperature. (**a**) Pt/WO_3_ powder XRD spectra. (**b**) H_2_ response measured at 800 nm.

The XRD patterns of Pt/WO_3_ powder samples, for which the precursor solution was dried at 393 K and then heat treated at 573–773 K for 1 h, are shown in Figure 5a. The Pt:W atomic ratio of the samples was set to 1:13. The diffraction peak assigned to WO_3_ in the 573 K sample was located at approximately 23°. The diffraction intensity increased with heat treatment temperatures. This is due to the improvement in crystallinity of WO_3_ and grain growth at higher temperatures, as shown in Figure S1 (Supplementary Materials). Moreover, multiple diffraction peaks were observed at higher treatment temperatures. Depending on the heat treatment temperature, WO_3_ can exist stably in various forms at room temperature, including monoclinic, triclinic, hexagonal, orthorhombic, or cubic systems. The intensity ratios of 022, 202, and 220 planes at approximately 23° were different for the three treatment temperatures. Compared to the International Centre for Diffraction Data cards (01-071-0131, 01-072-0677), the prepared Pt/WO_3_ powders seemed to be mixtures of monoclinic and orthorhombic phases for treatment temperatures up to 773 K. At higher temperatures, the diffraction intensity increased and the full width at half maximum of the peak at approximately 40° (derived from the Pt 111 plane) decreased. This decrease is due to the grain growth in Pt particles induced by the heat treatment at high temperatures. The crystallite sizes of Pt treated at 673 and 773 K, for which clear peaks were observed, were estimated using Scherrer’s equation. In the calculations, the peaks of the 111 plane were used for Pt. Based on Langford and Wilson’s empirical rule, the Scherrer’s constant was selected as a spherical approximation for WO_3_ and a cubic approximation for Pt [21]. The crystallite sizes of Pt at 673 and 773 K were 1.09 and 1.32 nm, respectively. These results indicate that the grain growth of Pt also occurred as the heat treatment temperature increased.

The transmittance and color changes of the Pt/WO_3_ powder samples heat treated at 573–773 K for 1 h are shown in Figures S4a and S5b (Supplementary Materials). The maximum color change was observed in the dispersed membrane prepared via heat treatment at 573 K, followed by the membranes treated at 673 and 773 K. The degree of color change at 473 K was smaller than that at 673 K (data not shown). In addition, the coloring reactions of the films dispersed with the 573 K and 673 K heat-treated powders saturated but did not reach saturation in the membrane dispersed with the 773 K heat treated powder. The maximum coloring rate was obtained for the film dispersed with the 573 K heat-treated powder (Figure S4b in Supplementary Materials). These results indicate that the optimal heat treatment condition for Pt/WO_3_ dispersed in the PDMS film with respect to the gasochromic reaction performance when hydrogen gas was 573 K. At this temperature, a WO_3_ crystal structure with coloring sites could be formed while retaining a large accessible surface area.

### 3.4. Hydrogen Gas Detection Performance of Pt/WO_3_ Composite Membranes

**Figure 6:**
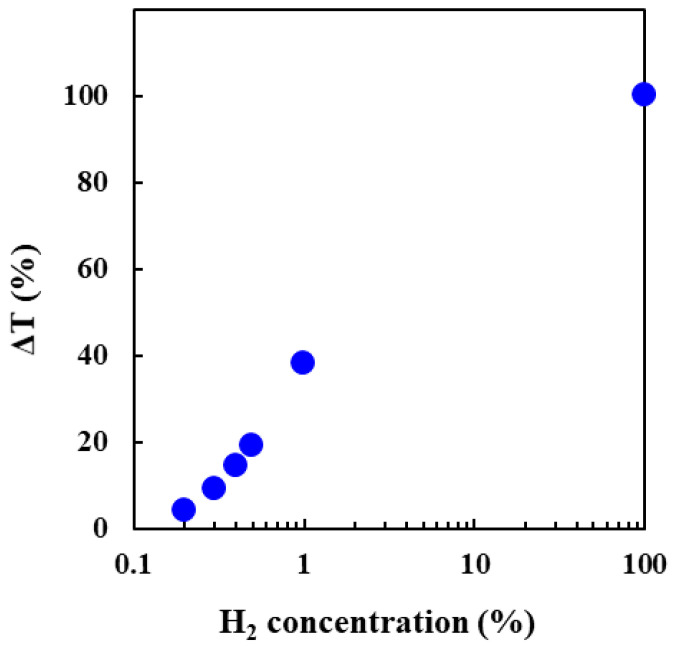
H_2_ gas detection performance of the Pt/WO_3_ composite membrane. (Pt:W = 1:13; heat treatment at 573 K; particle concentration of 0.75 wt.%; measurement wavelength of 800 nm; exposure to H_2_ gas concentrations of 0.1–100 vol%).

The H_2_ gas detection performance of the Pt/WO_3_ composite membrane is shown in Figure 6. The absorption was instantly saturated for 100% H_2_ gas, but for hydrogen concentrations less than 1.0%, the absorption did not saturate. The concentration gradient is the driving force for gas diffusion in the membrane. The opposite surface, which served as the target for the hydrogen gas flow, was exposed to the atmosphere, and the decolorization reaction occurred through the diffusion of oxygen from the atmosphere. Therefore, the color of the film is determined by the balance between the coloring and decoloring reactions. The latter was predominant at low hydrogen concentrations, which resulted in the absence of transmittance changes. Because 10% of ΔT is sufficient to determine the color change with the naked eye, the lower limit of detection was defined as 10%, and the concentration was 0.4%. Because hydrogen gas becomes explosive when its concentration exceeds 4% in the atmosphere, the lower limit of detection is one-tenth of the concentration, and the ability to detect hydrogen gas is sufficient for practical applications.

**Figure 7:**
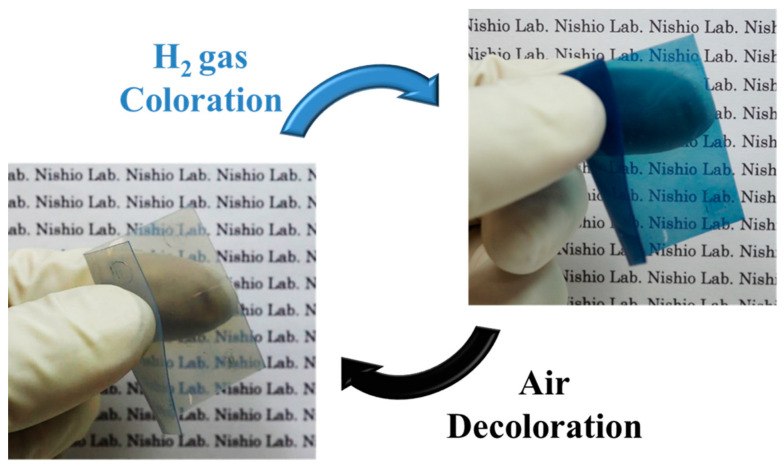
Hydrogen gas responsivity, transparency, and flexibility of the Pt/WO_3_ particle-containing membrane. (Pt:W = 1:13; heat treatment at 573 K; particle concentration of 0.75 wt.%; exposed to the H_2_ gas concentration of 100%).

As shown in Figure 7, the Pt/WO_3_ composite membrane is transparent and flexible. It can be easily attached to various shapes for practical applications. Using this membrane, the detection of hydrogen gas is qualitative, but the existence can be determined with the naked eye without the use of electricity. The Pt/WO_3_ nanoparticles exist not only on the surface of the membrane but are also distributed and exhibit a color change throughout the membrane. Therefore, they are expected to be resistant to aging degradations. The effect of humidity on Pt/WO_3_ coloration was verified in a previous study [22]. In this study, PDMS is a hydrophobic polymer, which prevents water from diffusing into the membrane, and the effect of humidity is considered to be small. However, water molecules are also generated during Pt catalysis in the decoloration step. This is because the oxygen molecules react with hydrogen in WO_3_ to return to the transparent state. The interference of the generated H_2_O on hydrogen gas sensing can be removed by heating the sensor over 353 K. For practical applications, the evaluation of the durability of repeated coloration/decoloration cycles might be needed in future.

## 4. Conclusions

Flexible and transparent Pt/WO_3_ nanoparticle-dispersed membranes toward H_2_ gas leakage sensor was prepared. First, the effect of the composition ratio of platinum and tungsten oxide was first investigated. The Pt:W = 1:13 membrane was found to be highly transparent and exhibited a sufficient color change in response to the H_2_ gas. The effects of the Pt/WO_3_ nanoparticle content in the composite membrane were also investigated. Although a simple trade-off exists between the nanoparticle content and transparency, the latter affects the hydrogen gas detection performance. Therefore, the content was optimized for the color difference. The membrane containing 0.75 wt.% of Pt/WO_3_ nanoparticles exhibited a local maximum color difference. Subsequently, the effect of heat treatment temperature was investigated. While high-temperature heat treatment increased the crystallinity of the Pt/WO_3_ nanoparticles, it also decreased the area accessible to hydrogen gas, contributing to a gasochromism reaction because of the grain growth in the particles. Sensitive detection and rapid response to H_2_ gas were achieved in the membrane dispersed with the 573 K heat-treated nanoparticles. Finally, sufficient visibility to the low concentration of H_2_ gas (0.4%, which is one-tenth of the minimum explosive concentration) and the flexibility of the membrane were confirmed. 

The membrane developed in this study is an inexpensive, safe, and easy-to-use hydrogen gas sensor. Because Pt/WO_3_ nanoparticles are dispersed in the membrane, resistance to aging degradation and abrasion can be expected. In situations where it is not possible to inspect the H_2_ gas-detection membrane immediately, it would be desirable for the coloring to remain after the detection of H_2_ gas. The membrane developed in this study returned to its original transparent color (from the blue color corresponding to the detection of H_2_ gas) relatively rapidly after it was placed in an air environment. A material that combines the hydrogen gas detection performance with memory properties, without compromising the advantages of the membrane, is a promising development. In future studies, the formation of a double-layer film with a gas barrier on one side or a controlled polymer network structure with selective H_2_ gas permeation will be investigated as possible solutions for achieving this goal.

## Data Availability

Not applicable.

## Supplementary Materials

The following supporting information can be downloaded from https://www.mdpi.com/article/10.3390/membranes12030291/s1, Figure S1: SEM images of Pt/WO_3_ nanoparticles. (a) Pt/WO_3_ nanoparticles prepared with different Pt contents. (b) Pt/WO_3_ nanoparticles prepared at different temperatures and the nanoparticle size distribution; Figure S2: Transmittance of sample membranes. The solid lines denote the transmittance in atmospheric air and the dotted lines, the transmittance in 100% H_2_; Figure S3: Dependence of transparency and H_2_ response on content percentage of Pt/WO_3_ nanoparticles. (a) Transmittance of sample membranes. (b) Transmittance when the films were exposed to 100 % H_2_ gas measured at 800 nm. The solid lines denote the transmittance in atmospheric air and the dot-ted lines, the transmittance in 100% H_2_; Figure S4: Dependence of H_2_ response on Pt/WO_3_ nanoparticle heat treatment temperature. (a) Transmittance of sample membranes. The solid lines denote the transmittance in atmospheric air and the dotted lines, the transmittance in 100% H_2_. (b) Maximum coloring velocity when the films were exposed to 100 % H_2_ gas.
