# Supplementary material for: Pt/WO3 Nanoparticle-Dispersed Polydimethylsiloxane Membranes for Transparent and Flexible Hydrogen Gas Leakage Sensors
Source: Membranes (Basel). 2022 Mar 2;12(3):291. doi: 10.3390/membranes12030291 (PMC8953145; doi:10.3390/membranes12030291)
Supplement: Supplementary file 1 [file membranes-12-00291-s001.zip › membranes-1584524-supplementary.pdf]

membranes

MDPI

Supporting Information

Pt/WO_3 Nanoparticle-dispersed Polydimethylsiloxane Membranes for Transparent and Flexible Hydrogen Gas Leakage

1. Scanning electron microscope observation of Pt/WO_3 nanoparticless prepared with various Pt contents and heat treatment temperatures

The FE-SEM images of Pt/WO_3 particles prepared with various Pt contents and heat treatment temperatures are shown in **Figure S1**. Each sample was prepared by evaporating the solvent of a dispersion obtained by bead milling a powder which was prepared in the same manner as the powder used for the XRD measurements. Because the particle size was larger than the calculated crystallite size, the diameter of the secondary particles that resulted from the aggregation of primary particles could be measured by FE-SEM.

1.1. Effect of Pt content on particle size

A smaller average particle size was observed in the Pt:W = 1:13 sample compared to the Pt:W = 1:100 sample (**Figure S1a**). The smaller particle size may be due to the presence of Pt particles, which promoted the heterogeneous nucleation of WO_3 particles during heat treatment and suppressed the grain growth of WO_3.

1.2. Effect of heat treatment temperature on particle size and distribution

The particle size increased with the heat treatment temperature (**Figure S1b**). The preparation of WO_3 particles using the sol-gel method consisted of the formation of tungsten alkoxide and dehydration condensation; in other words, thermal energy was used for the crystallization or grain growth of WO_3. Peaks originating from WO_3 were observed in the XRD patterns shown in **Figure 5a** for the heat treatment temperature of 673 K, indicating that the energy was mainly used for crystallization at up to 573 K during the heat treatment, and that the heat energy in the treatments above 673 K was used not only for crystallization but also for grain growth.

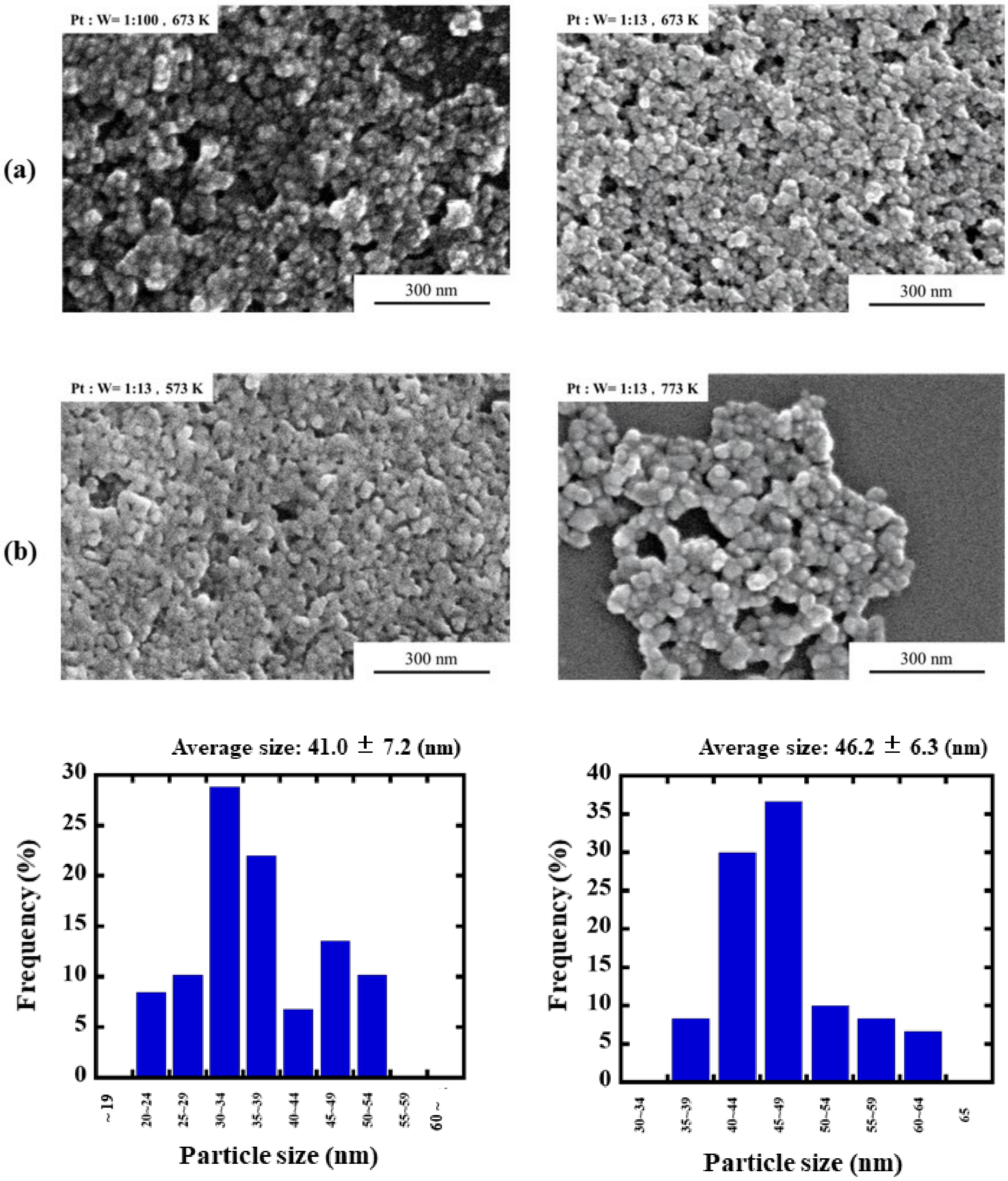

Figure S1. SEM images of Pt/WO_3 nanoparticles. (a) Pt/WO_3 nanoparticles prepared with different Pt contents. (b) Pt/WO_3 nanoparticles prepared at different temperatures and the nanoparticle size distribution.

2. Transmission spectra of the sample membranes exposed to 100% H_2 gas

The transmission spectra of the sample membranes exposed to 100% H_2 gas are shown in **Figure S2**. The transmittances of both membranes in the short wavelength range of 400–600 nm were reduced in atmospheric air (solid line). Because the band gap of WO_3 is about 2.6 eV (Wenderich, K. *et al.*, *Chem Rev.* 2016, 116, 14587) and WO_3 absorbs only light with wavelengths below 400 nm, the reduction in transmittance was attributed to wavelength-dependent Rayleigh scattering at the PDMS and WO_3 particle interfaces. There was no reduction in the transmittance in the visible region at 800 nm due to Rayleigh scattering, and the membrane with the higher platinum loading (Pt : W = 1 : 13) showed a larger transmittance change in response to hydrogen gas because of the atomization of the hydrogen molecules by the platinum impregnated on WO_3.

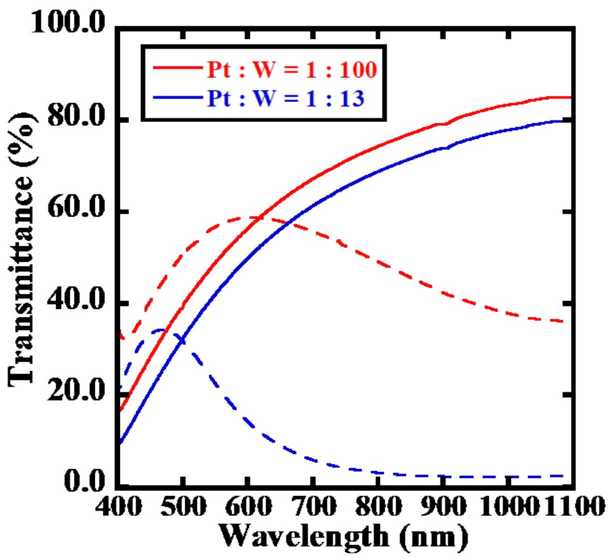

Figure S2. Transmittance of sample membranes. The solid lines denote the transmittance in atmospheric air and the dotted lines, the transmittance in 100% H_2.

3. Transmittance and Transmission spectra of the membranes

(a). The transmittance decreased with the increase in the number of dispersed particles because of Mie scattering due to the particle aggregates dispersed in the film. The transmittances of the 0.5 wt% and 0.75 wt% films were almost equal. This implies that the particle sizes in the aggregates in the two films were almost the same.

(b). The transmission spectra of membranes exposed to synthetic air and 100% H_2 gas are shown in **Figure S3(b)**. The transmittance of the membranes in the atmosphere, which are shown as solid lines, decreased with increasing amounts of dispersed particles at all wavelengths. This is because the absorbance increase with an increase of Pt/WO_3 amount in the membranes along with Lambert-Beer's law. In addition, there was a sharp reduction in the transmittance in the short-wavelength range of 400 to 600 nm. The starting point of the reduction shifted to longer wavelengths as the amount of dispersed particles increased. This reduction was caused by wavelength-dependent Rayleigh scattering at the interfaces between the particles and PDMS. Based on the observed light scattering and the absence of visible particles even in the membrane with the highest particle content (3.00 wt%), the size of the aggregates could be assumed to range from 100 nm to several μm. The transmittance of all membranes exposed to 100% H_2 gas, which are indicated by the broken lines, drastically decreased at the wavelengths of 800 to 1100 nm. The prepared membranes therefore showed a clear coloration change in response to hydrogen gas. In addition, the coloration response was accentuated in the membranes containing larger amounts of particles simply because of the larger numbers of particles that contributed to the coloration.

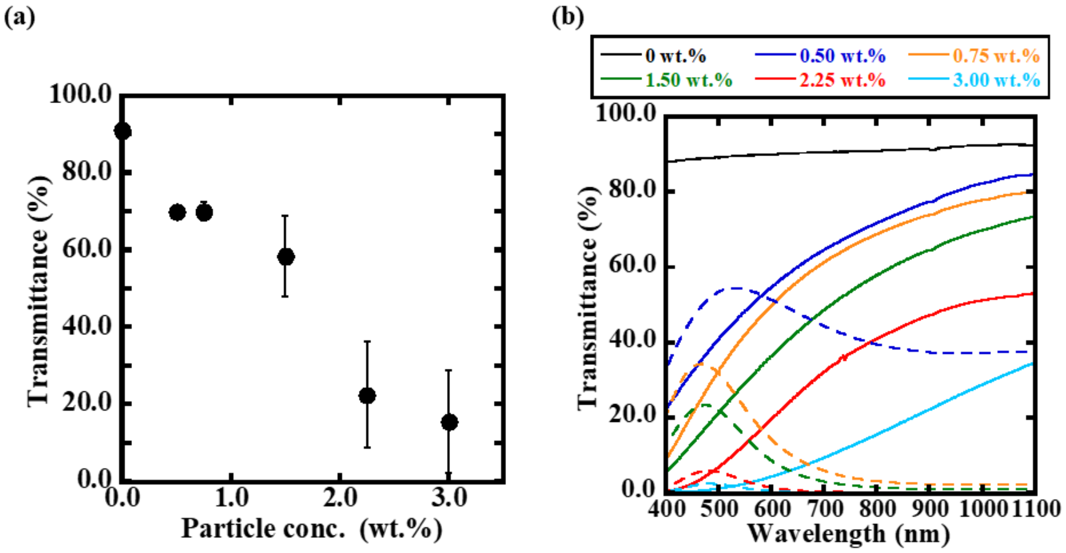

Figure S3. Dependence of transparency and H_2 response on content percentage of Pt/WO_3 nanoparticles. (a) Transmittance of sample membranes. (b) Transmittance when the films were exposed to 100 % H_2 gas measured at 800 nm. The solid lines denote the transmittance in atmospheric air and the dotted lines, the transmittance in 100% H_2.

4. Transmittance and coloring rates of the membranes

(a). The transmittance of the Pt/WO_3 powder samples heat-treated at 573–773 K for 1 h is shown in **Figure S4(a)**. All the film samples exhibited increased light adsorption at longer wavelengths in response to 100% hydrogen gas. At 800 nm, which is the longest wavelength in the visible light region, transmittance changes of at least nearly 50% were obtained in all the samples. In particular, absorption saturation was observed at the heat treatment temperatures of 573 and 673 K. In the gasochromism, protons enter the WO_3 lattice. To compensate the charge of the inserted protons, the nearby W^{6+} capture electrons and are reduced to W^{5+} with the adsorption of light. The electrons cause d-d transition with W^{6+} in the vicinity. In addition, the insertion of protons and electrons lead inter valence charge transfer between W^{5+} and W^{6+}. Although the crystallization of WO_3 at 573 K was so weak judged from the XRD pattern (**Figure 5a**), the formation of coloring sites was confirmed in these results for all the samples, including the one treated at 573 K. The samples treated at lower temperatures exhibited a better coloring response to hydrogen gas.

(b). The coloring rates of the films are shown in **Figure S4b**. The maximum coloring rate was obtained in the film dispersed with the 573 K heat-treated powder, followed by the membranes dispersed with the 673 K and 773 K heat-treated powders. The coloring change and coloring rate were the smallest in the film dispersed with the 773 K heat-treated powder, which had the highest crystallinity and the largest number of coloring sites. This is because the advanced grain growth of the Pt and WO_3 particles after the 773 K heat treatment resulted in a smaller accessible area that contributed to the gasochromism reaction with hydrogen gas compared to the powder heat-treated at 673 K (**Figire S1**). The specific surface area of WO_3 decrease by the grain growth with high temperature heat-treatment. Besides, protons and electrons must diffuse into the center part of WO_3 grain to complete the gasochromic coloration when the WO_3 grains became relatively large. The bulk diffusion is quite slower than surface diffusion in WO_3 crystal. Therefore, it was considered that the bulk diffusion of protons and electrons were rate determining step in gasochromic coloration of membrane dispersed with the 773 K heat-treated powder.

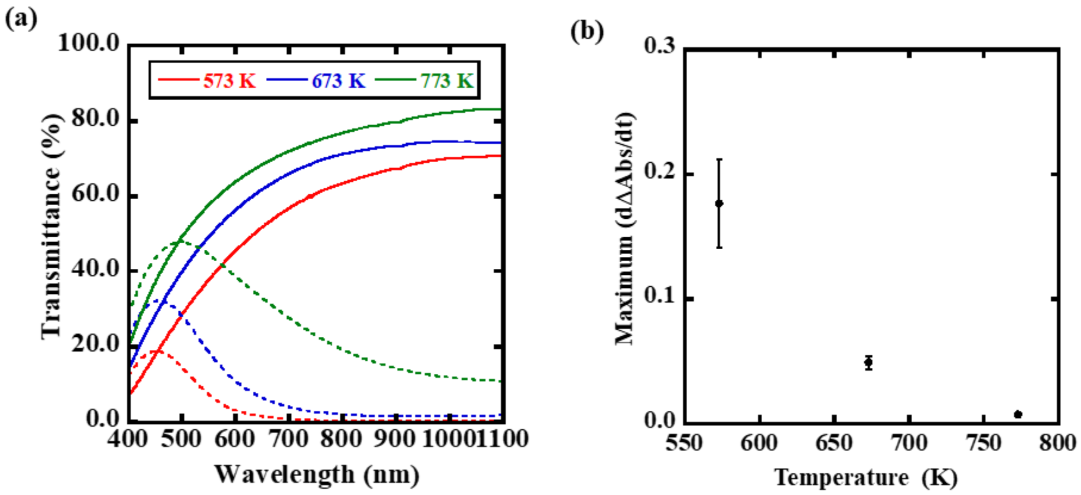

Figure S4. Dependence of H_2 response on Pt/WO_3 nanoparticle heat treatment temperature. (a) Transmittance of sample membranes. The solid lines denote the transmittance in atmospheric air and the dotted lines, the transmittance in 100% H_2. (b) Maximum coloring velocity when the films were exposed to 100 % H_2 gas.
